# Mandibular implant-assisted removable partial denture - Kennedy Class I to Class III modification – Case series with masticatory performance and satisfaction evaluation

**DOI:** 10.4317/jced.59777

**Published:** 2023-01-01

**Authors:** Rafael Araujo, Karla Zancopé, Rodrigo Moreira, Talita Barreto, Flávio Neves

**Affiliations:** 1 Doctorate Student of Oral Implantology. School of Dentistry, Federal University of Uberlandia, Minas Gerais, Brazil; 2 DDS, Ms, PhD. Professor of the Department of Occlusion, Prosthesis and Dental Material, School of Dentistry, Federal University of Uberlandia, Minas Gerais, Brazil; 3 Master Degree student of Oral Implantology. School of Dentistry, Federal University of Uberlandia, Minas Gerais, Brazil; 4 Postgraduate student, Department of Oral Implantology, Núcleo de Ensino e Estética em Odontologia, Itabuna, Bahia, Brazil; 5 DDS, Ms, PhD. Professor of the Department of Occlusion, Prosthesis and Dental Material, School of Dentistry, Federal University of Uberlandia, Minas Gerais, Brazil

## Abstract

In this work, we present 5 cases of Kennedy Class I patients with atrophic posterior mandible treated with the placement of 01 short WS Neodent® implant and a healing screw to support the removable prosthesis, transforming them into Kennedy Class III patients. To quickly evaluate and verify the benefit of this treatment, masticatory performance was evaluated with maximum bite force and chewing ability. A VAS questionnaire was also applied for a practical preoperative and postoperative evaluation of overall quality of life-changing for the patient after this treatment. This treatment plan was planned in order to reduce drastically the treatment costs and morbidity, and to enhance oral function and the quality of life for these patients. Also, this treatment lead to residual bone preservation, enhanced masticatory function and patient satisfaction. Especially in countries with a large number of patients with missing teeth and socio-economic difficulties to be fully rehabilitated with dental implants and fixed prosthesis treatment options with reduced costs are important to be in our armamentary os possibilities.

** Key words:**Dental implants, masticatory performance, chewing, oral function, mixing ability.

## Introduction

It is still very common, especially in countries where there is social inequality, to have a high prevalence of people with tooth loss (1,2). In this scenario, the need for prosthetic oral rehabilitation with fixed or removable prosthesis is enhanced (1,2). Some studies show that the percentual of elderly will continue to enhance until 2040, and as consequence and surrounded by social, cultural, and economic factors, a larger number of total or partially edentulous patients will also be increased (1,2).

Tooth loss, especially the posterior teeth, may cause a disturbance in the stomatognathic system, affecting sensorial and motor aspects that may interfere with the masticatory process (3-7). Partially edentulous patients may change their nutritive patterns by chewing limitations, which can lead to negative health and nutritional issues, and affect their quality of life (3,5). When the posterior teeth are lost, it is common for the patients to seek more soft foods, usually composed of an excess of carbohydrates and lowered in fruits, vegeTables, proteins, and nuts, which consequently makes a less nutritive diet (6,8,9). To overcome the absence of the posterior teeth and recompose the aesthetic and masticatory function, a removable partial prosthesis (RPP), fixed partial prosthesis, or implant retained prosthesis are recommended (6,7,10,11).

The masticatory function may be evaluated through maximum bite force, masticatory performance, or chewing/mixing ability. These methods, which are used to evaluate the masticatory function, have gained great popularity in the latest years, evaluating and comparing treatments and their impact on the quality of life, chewing, and trying to project nutritional aspects for the patients (4,6,11).

Mandibular posterior bone atrophy may lead patients to a series of limitations of treatment options due to the consequences of low bone quality, and often insufficient height and width of residual bone, superficialization of the inferior alveolar nerve, and altered or increased occlusal dimension (7). For those reasons, when an implant oral rehabilitation is proposed, it is often necessary to initiate with previous reconstructive surgeries. In these cases, we come across some sensitive techniques subjected to a series of complications. Onlay and inlay autogenous bone grafts, guided bone regeneration, split crest technique, alveolar bone distraction, and inferior alveolar nerve lateralization are some of the most cited options in the literature, each of them with their own disadvantages and complications associated (7,8,12). All these procedures have in common the need for an experience of the surgeon, as well as an increased cost, time of treatment, and morbidity for the patient (12). An excellent treatment alternative for the atrophic posterior region is the use of short implants (7).

An RPD is a treatment associated with a reduced total cost that may replace several teeth and have a general increase in the patient chewing function (4). Nevertheless, patients with RPD have a decrease in their masticatory function when compared to fixed treatment options (8-10). In Kennedy Class I patients treated with RPD, due to the absence of support in posterior teeth, this treatment is reported to presented low retention and stability making chewing difficult and producing pain in the mucosa that is compressed when chewing is taking place (8,14). This treatment is associated with overall dissatisfaction and oral discomfort by the patient in approximately 60% of the cases, and many abandon the use of this prosthesis (8,14). Almost 40% of the partially mandibular edentulous patients are classified as Kennedy Class I (15). Other issues such as increased carious lesions and periodontal disease in the pillar tooth are frequently observed (14,15).

In this work, we present 5 cases of Kennedy Class I patients with atrophic posterior mandible treated with the placement of 01 short WS Neodent® implant and a healing screw to support the removable prosthesis, transforming them into Kennedy Class III patients. To quickly evaluate and verify the benefit of this treatment, masticatory performance was evaluated with maximum bite force and chewing ability. A VAS questionnaire was also applied for a practical preoperative and postoperative evaluation of overall quality of life-changing for the patient after this treatment. This treatment plan was planned in order to reduce drastically the treatment costs and morbidity, and to enhance oral function and the quality of life for these patients.

## Case Report

**Figure 1:**
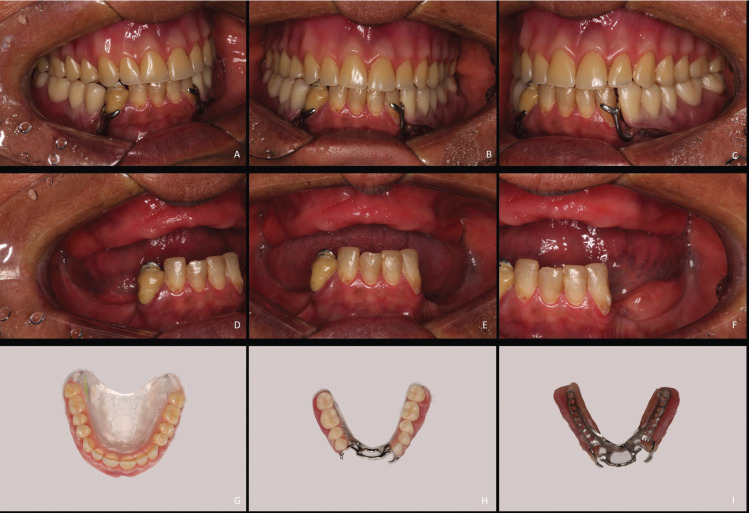
Preoperative intraoral photographs. A, B, C. Preoperative intraoral photographs using upper complete denture and lower partial denture. D, E, F. Frontal and lateral intraoral photograph without the removable dentures showing residual ridge depth and bone atrophy. G, H, I. Photographs of the upper complete denture and lower partial denture used by the patient before implant placement.

**Figure 2:**
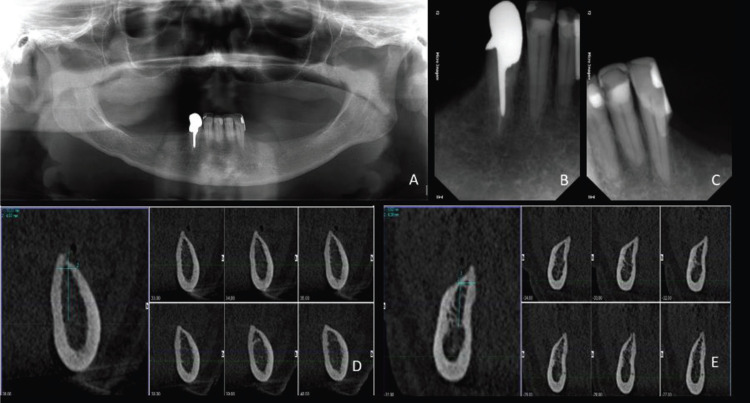
Preoperative radiographic and tomographic images. A. Preoperative panoramic radiography. B and C, Periapical radiographs of the pillar teeth for the removable prosthesis, the right canine, and left lateral incisor. D, E. Computed tomography of the regions planned for implant placement. It is possible to see the bone atrophy on the mandibular posterior region on both sides.

All these 5 cases reported followed exactly the same protocol. All surgeries were performed by the same surgeon (RZA). All patients were complete maxillary edentulous and mandibular Kennedy Class I (Fig. 1). If the total removable superior prosthesis and partial inferior prosthesis were not suiTable, a new pair of removable prosthesis were accomplished before implant surgery. A common complaint in all cases was some sort of dissatisfaction with the use of the inferior RPD, usually related to pain when chewing, prosthesis instability, or general discomfort. All patients had severe bone atrophy with indication of reconstructive surgery in the anterior (next to the pillar teeth) and/or posterior mandibular region if a complete implant planning surgery was the main treatment option (Fig. 2).

The impossibility to bear the costs of a complete implant treatment associated or not with reconstructive surgery was a common issue for all of these cases, making this treatment option unavailable. Alternatively, aiming for a significant overall reduction of treatment costs with a treatment that would allow patients to use their RPD with increased comfort, stability, and less mucosal compression and pain during chewing, it was proposed to place short implants bilaterally in the posterior region. The option to use healing screws and not to perform prosthesis implant crown goes in the same direction for cost reduction, whereas the patient would have an additional cost for the crown and for the RPD adaptation or replacement for a new one.

**Figure 3:**
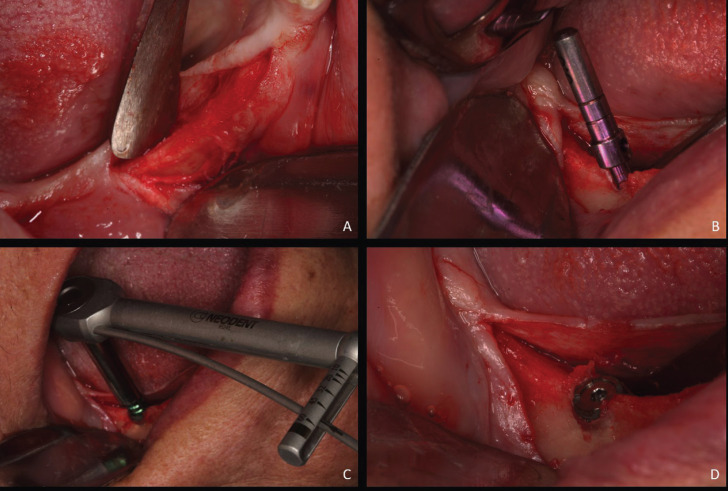
Implant surgery placement. All surgeries followed the same protocol. A. Alveolar ridge incision and periosteal tissue detachment. B. After the 2.0 drilling, a parallelizer was placed to check the correct tridimensional position of the implant to be inserted. C. Implant engaged and torque measuring. D. Implant installed with a cover screw to be reopened in 4 months.

All surgeries followed the same protocol, and were accomplished in the same dental office (Dental School of the Federal University of Uberlandia). After Lidocaine 1:100.000 local anesthetics were accomplished, a small crestal incision and periosteal elevation was made only on the region of implant placement. If the last remaining teeth were the 1º or 2º Pre-molar, implants would be placed in the 2º molar region. If the last remaining pillar teed were the canines or anterior, the implants would be installed in the 1º molar region. All implants were Neodent® WS short implants, with 4mm width and 5 or 6mm height. All surgeries were executed with the assistance of parallelizer pins to help to guide the 3-dimensional implant angulation and the occlusal patient reference (Fig. 3).

**Figure 4:**
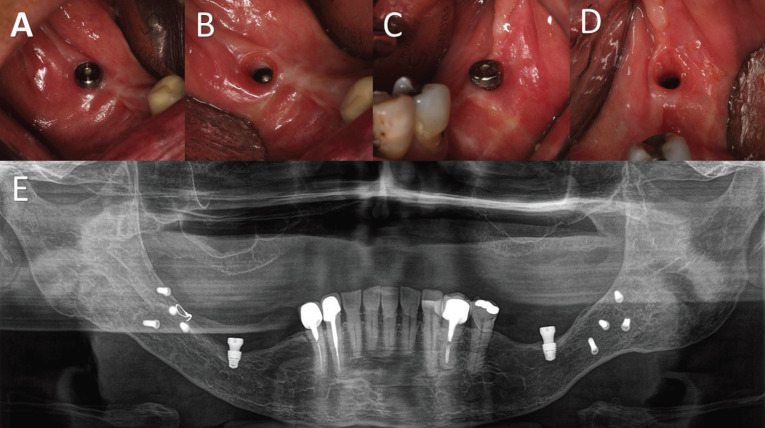
In the follow-up periods, if the healing screw were more than 1mm above the soft tissues, they were changed to a smaller one. A, C. Replacement of the healer on the right side and left side respectively. B, D. Soft tissue aspect without the healer bilaterally. E. Panoramic postoperative radiography from the same patient showing the Neodent WS short implant and healing screw. The screws in the mandibular angle are from the rigid internal fixation of a previous orthognathic surgery.

All patients were submitted to a 2-time protocol, and a period of 4 months was waited for the osseointegration period before reopening the implants. After 1 to 2 weeks after healing screws were installed, sutures were removed and the patient initiated the use of their RPD over the implant/healing screw. Follow-up revisions were each 15 days in the first 2 months and then monthly until the sixth month. After that, patients were placed on a regular follow-up schedule, with 2 visits per year or before that if any issue would arise. During the follow-up appointments, if necessary, adjustments were made in the RPD and a substitution in the healing screw was accomplished so it could remain at a 0.5mm or maximum 1mm above the gingival tissue (Fig. 4).

Before implant surgery and 6 months after healing screw placement, all patients were evaluated for a satisfaction survey with a VAS (Visual analogue scale) questionnaire and for the masticatory function with a maximum bite force and mixing ability test. All these tests and questionnaires are easy to perform, quick, validated by the literature, and reliable to evaluate treatment outcomes. The maximum bite force was evaluated with the use of a Gnatodynamometer. The patient bites 5 times on the right side and the highest and lowest result was discarded. The other 3 were made an average to obtain the final result. Masticatory performance was evaluated through chewing gum and specific software developed to analyze the mixed gum. The Viewgum software (ViewGum© software, dHAL Software, Greece, www.dhal.com) was specifically developed to evaluate mixing ability from the digital image obtained through photographing or scanning two-colored chewing gums (21,22,26). This methodology is widely used for masticatory performance evaluation purposes in the literature (21,22,26).

All the results of the VAS were positive for all patients. Maximum bite force increased in all cases and masticatory performance was also enhanced for all 5 patients (Table 1, Table 1 cont.). With 1 year of follow-up, no patient has had any major complaints or implant loss. Only 2 cases of healing screw loosening happened and were solved with regular appointment and clenching. In follow-up appointments healing screws were detached, polished, and torqued again, and if RPD adjustments were necessary, they were accomplished.

**Table 1:**
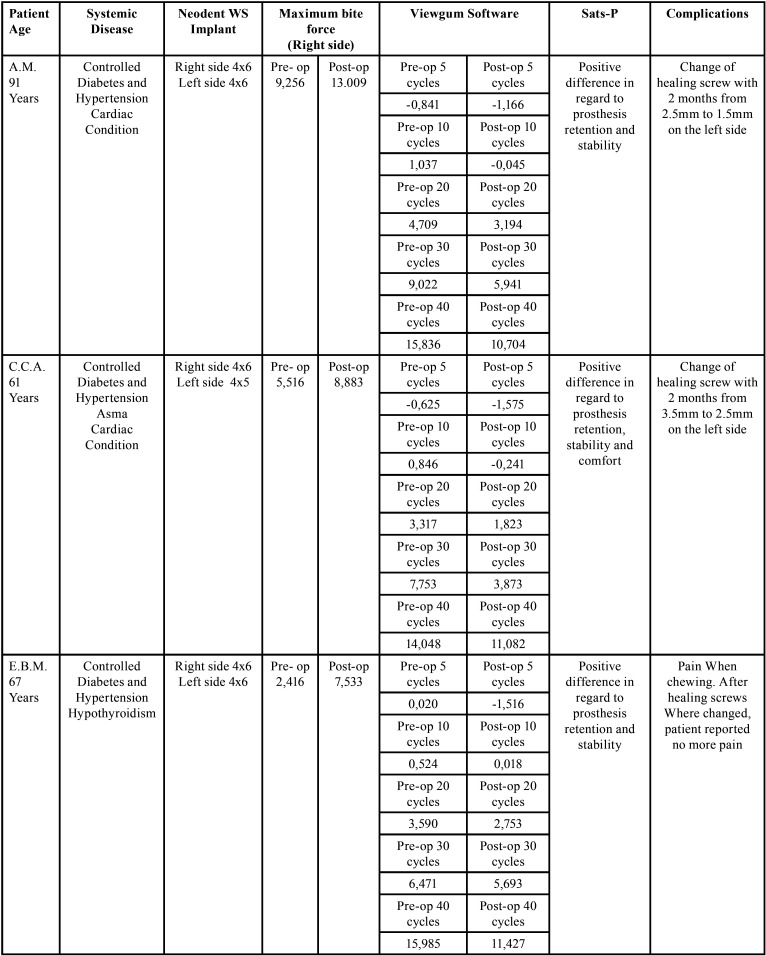
Data from the 5 consecutive patients.

**Table 1 cont.**
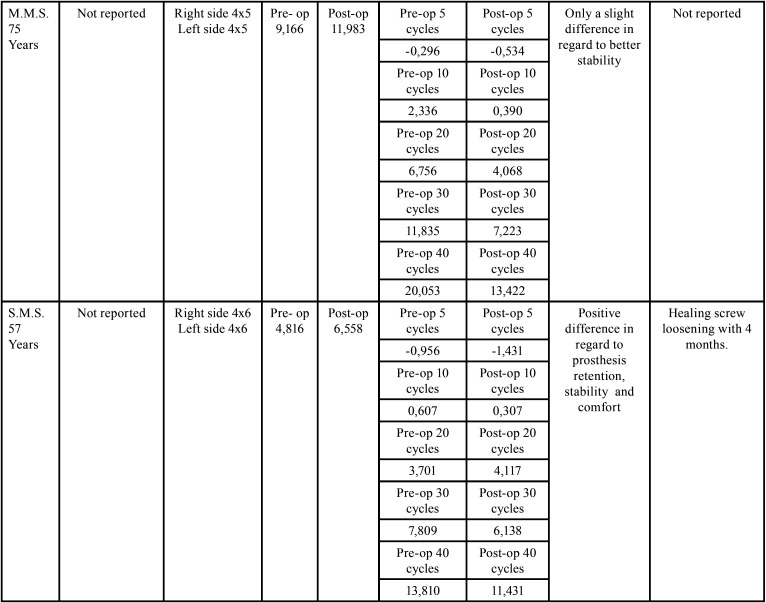
Data from the 5 consecutive patients.

## Discussion

Kennedy Class I patients, but mostly in any other patient with several tooth losses, problems with chewing impairment, muscular disturbance, and decreased nutrition and quality of life may be a negative consequence (5,8,17). The RPD for Kennedy Class I patients may lead to poor retention, stability, and ultimately abandonment of the prosthesis use. Nowadays, the importance of increasing or maintaining masticatory capacity is a favorable factor in healthy aging and preservation of some cognitive functions (18,19).

Placement of short implants to support bilateral free end mandibular prosthesis is being published by some papers in the last few years in the literature. This treatment has some advantages for the patient such as low treatment cost, preservation of the residual bone, reduced morbidity treatment option, better loading distribution in the pillar removable prosthesis teeth (and increased tooth survival), increased speech ability and masticatory function, better prosthesis stability and comfort during chewing, and ultimately, enhanced satisfaction and patient quality of life (9,12,13,20-24).

It has already been suggested that 3 masticatory units are sufficient to create a significative positive outcome in the masticatory performance of patients (short dental arch). In our cases reported, the maximum bite and mixing ability prove these improvements (22,24). Placement of implant, if in the 1º or 2º molar region, will depend n the remaining pillar teeth (22). The position of the short implant must be carefully planned to aim the support the RPD and even make it possible (if so desired), to the placement of additional implants for a fixed partial prosthesis. Systematic reviews demonstrated good survival rates for this type of treatment, varying between 91.7 - 100%, similar to other mandibular regions used exclusively to support implant fixed prosthesis (22-24).

We did not find any major complications in our case series. During the follow-up period it was necessary to replace some of the healing screws to adjust their height to be 0.5mm to a maximum of 01mm above the gingival soft tissue. Only 2 patients showed loosening of one of the healing screws before the scheduled appointment. Any other major complication in regard to peri-implant tissue or bone loss was not observed. Patients are followed with periapical radiographs. Other literature reviews of this type of treatment also do not report major complications as an issue to be concerned (22-24).

To evaluate the effectiveness of this technique we used a satisfaction questionnaire (SATS-PRO), which provides an estimative of the impact of the buccal conditions in edentulous patients (25). All patients had better results regarding their personal satisfaction after the treatment indicating enhancement of the quality of life, both physical and psychological. Another aspect evaluated was the masticatory performance to verify if the use of these short implants/healing screw would provide good support for the RPD with functional results. The use of a bicolor chewing gum provides a test of mixing ability and is capable to be analyzed by a specific developed software Viewgum®, which allows to establish through graphics and numerical results the masticatory efficiency through the mixing of the colors of the gum (26,27). The maximum bite force provides us objective numerical data on the chewing capacity of the patient, and if it is increased we expect that the patient may include in his/her diet harder and more consistent food, such as meat, nuts, fruits, and vegetables. Both masticatory performance tests showed improved results, as we can see in Table 1, Table 1 cont. Sats-Pro questionnaire also pointed to improvement in the satisfaction of the patient with the treatment after implant placement. All of these tests are easy to perform, fast, with low cost, and may be incorporated and applied in our daily routine in our offices. They are important tools to create treatment data, communicate to the patient, and also for legal purposes.

## Conclusions

Placement of short implants to support RPD in Kennedy Class I mandibular patients has several advantages such as low cost, residual bone preservation, low morbidity, better masticatory loading distribution, and enhanced masticatory function and patient satisfaction (10,13,14,21,22). Especially in countries with a large number of patients with missing teeth and socio-economic difficulties to be fully rehabilitated with dental implants and fixed prosthesis treatment options with reduced costs are important to be in our armamentary os possibilities. Also, patiens may not be able or do no want to perform complex reconstructive surgeries previous to dental implant placement. Although it has a series of limitations, this treatment may pose as a good alternative for patients with the profile describe in this papper. It is important to highlight that many of those patients are elderly, and a treatment that reduces morbidity and overall treatment time is always convenient.
